# Toward the Baltic Sea Socioeconomic Action Plan

**DOI:** 10.1007/s13280-019-01264-0

**Published:** 2019-10-11

**Authors:** Markku Ollikainen, Berit Hasler, Katarina Elofsson, Antti Iho, Hans E. Andersen, Mikołaj Czajkowski, Kaja Peterson

**Affiliations:** 1 Department of Economics and Management, University of Helsinki, P.O. Box 27, 00014 Helsinki, Finland; 2 Department of Environmental Science, Aarhus University, Frederiksborgvej 399, 4000 Roskilde, Denmark; 3 Department of Economics, Swedish University of Agricultural Sciences, Box 7013, 750 07 Uppsala, Sweden; 4 Biosociety Unit, Natural Resources Institute, Finland (Luke), Latokartanonkaari 9, 00790 Helsinki, Finland; 5 Department of Bioscience, Aarhus University, Vejlsøvej 25, 8600 Silkeborg, Denmark; 6 Faculty of Economic Sciences, University of Warsaw, Dluga 44/50, 00-241 Warsaw, Poland; 7 Stockholm Environment Institute Tallinn Centre, Lai 34, 10133 Tallinn, Estonia

**Keywords:** Cost-effectiveness, Incentives, Innovation, Manure, Performance-based policy

## Abstract

This paper analyzes the main weaknesses and key avenues for improvement of nutrient policies in the Baltic Sea region. HELCOM’s Baltic Sea Action Plan (BSAP), accepted by the Baltic Sea countries in 2007, was based on an innovative ecological modeling of the Baltic Sea environment and addressed the impact of the combination of riverine loading and transfer of nutrients on the ecological status of the sea and its sub-basins. We argue, however, that the assigned country-specific targets of nutrient loading do not reach the same level of sophistication, because they are not based on careful economic and policy analysis. We show an increasing gap between the state-of-the-art policy alternatives and the existing command-and-control-based approaches to the protection of the Baltic Sea environment and outline the most important steps for a Baltic Sea *Socioeconomic* Action Plan. It is time to raise the socioeconomic design of nutrient policies to the same level of sophistication as the ecological foundations of the BSAP.

## Introduction

The greatest environmental challenge in the Baltic Sea is eutrophication and the ecological risks it causes (Reusch et al. 2018). The Baltic Sea Action Plan (BSAP), accepted by HELCOM member countries in 2007 and revised in 2013, aims to achieve a good ecological status of the sea. It defines overall reduction targets of nutrients (15 200 t P and 118 000 t N) as well as specific targets for all sub-basins of the sea to be achieved by 2021 (HELCOM 2013). BSAP is based on innovative ecological modeling of the Baltic Sea, combining riverine loading and transfer of nutrients within the sea with eutrophication in the sea and its sub-basins (Backer and Leppänen 2008). Thus, the BSAP has a firm rooting in the knowledge of the dynamics of eutrophication in the Baltic Sea. However, the ecological goals of the BSAP are mechanically translated to nationally assigned reduction targets, without consideration of whether such an allocation is cost-effective or follows equity of cost-burden sharing.

Since the acceptance of the BSAP, overall nutrient loading to the Baltic Sea has slowly decreased—by approximately 19% for P and 24% for N—between the reference period of 1997–2003 and 2012–2014 (HELCOM 2017). The reduction targets of the BSAP are 11% for N and 43% for P for the riverine loads, so the distance to the goal is still considerable. The area of anoxic bottoms has been increasing, however (Carstensen et al. 2014). The slow progress in the implementation of the BSAP has some obvious reasons. In contrast to the science-based ecological modeling, the assigned country-specific reductions of nutrient loading have not reached the same level of sophistication. They have not been founded on economic and policy analysis. Instead of being cost-efficient, they are unnecessarily expensive and considered unfair, especially in regard to the country-specific allocation of cost-burden (Ollikainen and Honkatukia 2001; Gren 2008; Elofsson 2010; Hasler et al. 2014; Nainggolan et al. 2018; Reusch et al. 2018). The results of these studies suggest that there would be considerable cost savings if more of the nutrient emission reductions occurred in Poland and Russia. However, Finland and Sweden would receive most of the benefits from improved water quality (Ahtiainen et al. 2014). As a result, achieving a cost-effective solution requires multi-national thinking and hence faces various policy challenges.

Perhaps the largest hindrance in designing efficient paths for achieving the abatement targets at the time when BSAP was signed is the vague understanding of polluters’ incentives and difficulties in designing a policy that would take polluter heterogeneity into account. Since then, there has been considerable progress in research on policy instruments, incentives, and mechanisms, particularly in the context of water quality management (see Winsten and Hunter 2011; Xepapadeas 2011; Shortle 2017; Shortle and Horan 2017 for surveys). Despite this progress in knowledge, countries have rarely adopted innovative policies, such as using environmental benefit indexes, tendering systems or trading and compensation mechanisms. Furthermore, means to promote citizens’ engagement and awareness are lacking. Today, an increasing gap exists between the new policy possibilities and the existing policy structure in the protection of the Baltic Sea. The current policy is characterized by command-and-control-based approaches to point sources and technology-specific policies (command-and-control as well as agri-environmental support) toward nonpoint sources. It is time to raise the socioeconomic design of nutrient policies to the same level of scientific excellence as the natural science underpinnings of the BSAP.

The objective of this paper is to outline, at a strategic level, the most important steps to improve nutrient policies in the Baltic Sea region. We identify the largest weaknesses of current policies and suggest economic instruments that are better suited for regulating point and nonpoint sources of nutrient loadings. We emphasize the need for technological developments in nonpoint sources to reduce loading and analyze key factors needed to promote it. We demonstrate the need for coherence between water and emerging climate policies, especially in the case of agriculture. We point out the main obstacles to improving policies. We outline basic ingredients for an effective Baltic Sea Socioeconomic Action Plan. Such a plan is needed not only for national policymakers but also for the European Union’s policies relevant to combating eutrophication, such as the Common Agricultural Policy (CAP), environmental directives, and the Baltic Sea Region Strategy.

## Reduction potential versus abatement costs—Point and nonpoint sources

There is a fundamental difference between nutrient load policies aimed at point sources, such as waste water treatment plants (WWTPs) and industry, and those targeting nonpoint sources, such as agriculture and forestry. Point sources release nutrient loads via definite points, pipes, so their loads can be easily measured and directly subjected to regulation. Nonpoint loads, in contrast, are diffuse, coming from surface and drainage; they are stochastic due to varying weather and often associated with a delay between action and releasing loadings. Therefore, it is not possible to register the exact amounts of nutrients that are released from a given field parcel or forest plot, making it impossible to levy policy instruments directly on loads (Shortle and Dunn 1986). The only possibility is to target instruments on inputs and management practices that indirectly determine nutrient loads. Thus, for nonpoint sources, only a second-best policy is an option. Furthermore, while effective technologies can be employed to reduce loads from point sources, often at low costs, measures for agricultural nonpoint are less effective and sometimes more expensive and uncertain due to stochasticity and spatial heterogeneity in biogeochemical processes (Elofsson 2010; Hyytiäinen and Ollikainen 2012; Hasler et al. 2018). Therefore, cost-effectiveness analyses give much higher abatement rates for point sources than nonpoint sources. This feature is sometimes understood poorly; for instance, BSAP gives little attention to the possibilities and need of reducing nitrogen in WWTPs cost-effectively.

Table 1 provides information on nutrient inputs to the Baltic Sea apportioned to sources including natural background and atmospheric deposition on the sea. The table is based on annual reporting by the contracting parties to HELCOM (Personal communication Lars Svendsen, DCE, Aarhus University). Diffuse sources, mostly agricultural, are responsible for 39% of nitrogen and 49% of phosphorus loads, while the respective shares of point sources are 8% (N) and 16% (P).

**Table 1:** Nutrient loads (tons) to the Baltic Sea in 2000 and 2014 (Personal communication Lars Svendsen, DCE, Aarhus University). Figures are actual, nonclimate normalized loads. Point sources include point sources directly to the sea and point sources to inland surface waters (see also http://www.helcom.fi/baltic-sea-action-plan/nutrient-reduction-scheme/progress-towards-maximum-allowable-inputs)

Source/year	N	N	P	P	Reduction by 2014	
	2000	2014	2000	2014	N	P
Natural background	188 000	165 000	9 200	8 000	23 000	1 200
Point sources	72 000	58 000	8 400	4 400	14 000	4 000
Diffuse sources	434 000	293 000	20 300	13 800	141 000	6 500
Atmospheric deposition on BAS	310 000	240 000	2 100	2 100	70 000	0
Total	1004 000	756 000	40000	28 300	248 000	11 700

To illustrate the importance of the effectiveness of measures and their costs, consider cost estimates for nutrient reduction in Finnish agriculture: reducing both nitrogen and phosphorus loads by 20% entails marginal costs € 9,4 (kg N)^−1^ and € 223 (kg P)^−1^ (Hyytiäinen and Ollikainen 2012). Compare these estimates to marginal abatement costs in waste water treatment plants (WWTPs) for the Baltic Sea: a 90% reduction in nitrogen costs approximately € 11 (kg N)^−1^ and a 95% reduction in phosphorus costs € 17 (kg P)^−1^ (Hautakangas et al. 2014). The difference in marginal costs between the two sectors is large, especially in regard to the cost of phosphorus reduction. Adapting from Hautakangas et al. (2014), if WWTPs abate according to the Urban Waster Water Directive (UWWTPD), their annual abatement costs are less than 500 M €. Increasing the abatement rate up to 95% P and 90% N would increase their costs to approximately 1 100 M€ and produce a reduction of 85 000 t N and 9 600 t P (Hautakangas and Ollikainen 2018). Thus, requiring a high abatement rate in WWTPs and allocating the remaining part of the reduction target to agriculture would keep the total costs low. Following this approach, Ahlvik et al. (2014) cover both WWTPs and agriculture and suggest that in the cost-effective solution, the total cost of achieving the BSAP targets would in this case be approximately 2 000 M€. Hasler et al. (2014) estimate a total cost of 4 100 M€ for a similar solution. This range reveals some uncertainty on the costs and data. Nevertheless, following cost-effectiveness could lead to significant savings, relative to arbitrarily selecting targets for different measures.

This discussion provides a lesson: cost-effective abatement with equalization of the marginal abatement cost should be the guiding principle of nutrient policies toward point and nonpoint sources, because this principle reflects best the technological and economic possibilities to reduce loads the most.

### Policies for point sources

Abatement of nutrients in WWTPs and industrial point sources provides the backbone of any well-designed policy for the Baltic Sea protection. Abating both nutrients in the WWTPs is certain and less costly relative to other sectors, albeit reducing nitrogen requires a high initial but a long-lasting investment. The best available technique facilitates higher emissions reductions than the abatement rates in the above example, as experience in many countries has demonstrated. Hautakangas and Ollikainen (2019) provide examples of abatement rates in various plants. They suggest that it would be justified to require WWTPs to abate at least 95% of phosphorus and close to 90% of nitrogen.

The main weakness of nutrient policies toward point sources is that both the EU’s Urban Waste Water Directive and HELCOM recommendations are inattentive relative to current abatement possibilities and abatement costs, and we propose that they should be scaled up accordingly.

Another weakness of policies toward point sources is their reliance on command-and-control instruments only. Countries should make further efforts by financing research and providing result-based schemes, to create better incentives to extend abatement beyond conventionally known abatement technologies. These efforts include economic incentives not only to increase abatement but also to find new and innovative ways to treat wastewater, such as extracting phosphorus from sewage water for new products and using the abatement process and sludge to produce energy in the spirit of the EU’s circular economy initiative.

A topic not so often discussed in the literature is abatement in industrial point sources. It is a drawback that HELCOM does not report separate data on industrial point sources’ contributions to nitrogen and phosphorus loads at the Baltic Sea level but provides only an aggregate number for all point sources. Databases for improving water policies toward industrial point sources should be developed.

### Policies for agricultural nonpoint sources

As the setting of optimal taxes or quantitative limits on runoff from fields is infeasible, many countries have agri-environmental schemes that rely on farmers’ voluntary participation and pay for taking conservation measures among the given a set of measures targeting nutrient loads. Designing an effective voluntary agri-environmental program faces three basic challenges: (i) how to make it effective for water protection, (ii) how to invite the farmers that could contribute the most to the goals of the program (Schroeder et al. 2015), and (iii) how to ensure that farmers comply with the requirements (Winter and May 2001). Participation rates in programs depend on farmers’ attitudes toward a cleaner environment and the amount of the compensation relative to the costs and trouble of applying the measures (e.g., Pannell et al. 2006). Higher compensation increases participation, and more demanding and costly measures reduce it.

#### Voluntary agri-environmental programs

**Fig. 1:**
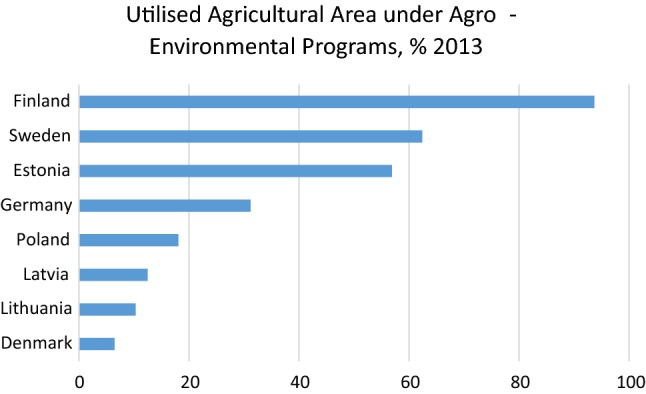
Participation rates in voluntary agro-environmental programs in percent of utilized agricultural area Source: Eurostat

All member states in the EU have a voluntary agri-environmental program that compensates for the average costs of implementing the measures using area-based payments. Furthermore, the Basic Payment Scheme (CAP Pillar I income support) contains cross-compliance conditions, which require farmers to undertake environmental measures to be eligible for the single farm payment. Figure 1 summarizes the participation of farmers in the agro-environmental schemes in 2013 (Source: Eurostat).

Participation rates in voluntary agri-environmental programs vary enormously, with Denmark, Poland, Latvia, and Lithuania having low participation rates. Caution should, however, be used when assessing the role of high participation rates in producing water quality improvement. There is a trade-off between participation and ambition. For instance, Lankoski and Ollikainen (2013) demonstrate that the Finnish agri-environmental program has been very generous, providing large overcompensation, keeping low profit farms and marginal lands in production, and actually increasing nutrient loading. Hasler et al. (in the present volume) find large heterogeneity among farm types in farmers’ reservation prices to enter into voluntary agro-environmental schemes.

The impact of the measures included in agri-environmental schemes on nutrient loads depends on their environmental effectiveness and local conditions. Most programs offer a set of measures for farmers to choose from, such as buffer strips and buffer zones, reduced fertilization, catch crops, conservation or restoration of wetlands, grassland management, set-aside and cover crops (Zimmerman and Britz 2016). Unfortunately, some abatement or conservation practices may have adverse effects on other environmental targets. For instance, a measure effective in reducing particulate phosphorus tends to increase the loading of dissolved phosphorus (Dodd and Sharpley 2016), or management designed for water quality may increase air emissions (Aillery et al. 2005; Smith et al. 2017).

We conclude that the major reason for the slow progress of nutrient abatement in agriculture is that current policy instruments are not cost-effective and are sometimes insensitive to trade-offs between environmental targets.

#### Incentives and targeting

The slow progress in reducing nutrient loading is related to the way the programs are tailored. The AES programs compensate farmers for taking the requested measures irrespective of their impacts on nutrient loads or other environmental effects. As a result, a farmer adopting a measure that leads to little improvement in water quality receives the same compensation as a farmer who can efficiently reduce nutrient loads. This is a clear waste of resources: both public funds and farmers’ efforts. A shift to performance-based schemes, drawing on the expected impacts of input choices on loads, would lead to environmental effectiveness, promote the best measures in each location and provide a higher return to public funds. There are two promising avenues to improve performance of policies: a shift from flat rate (cost-based) subsidies to incentive-based instruments, and increasing environmental targeting (result-based measures) by introducing environmental benefit indexes to both flat rate policies and incentive-based schemes. Both avenues facilitate improved environmental targeting, and the incentive-based instrument helps by using government budget money more efficiently.

**Fig. 2:**
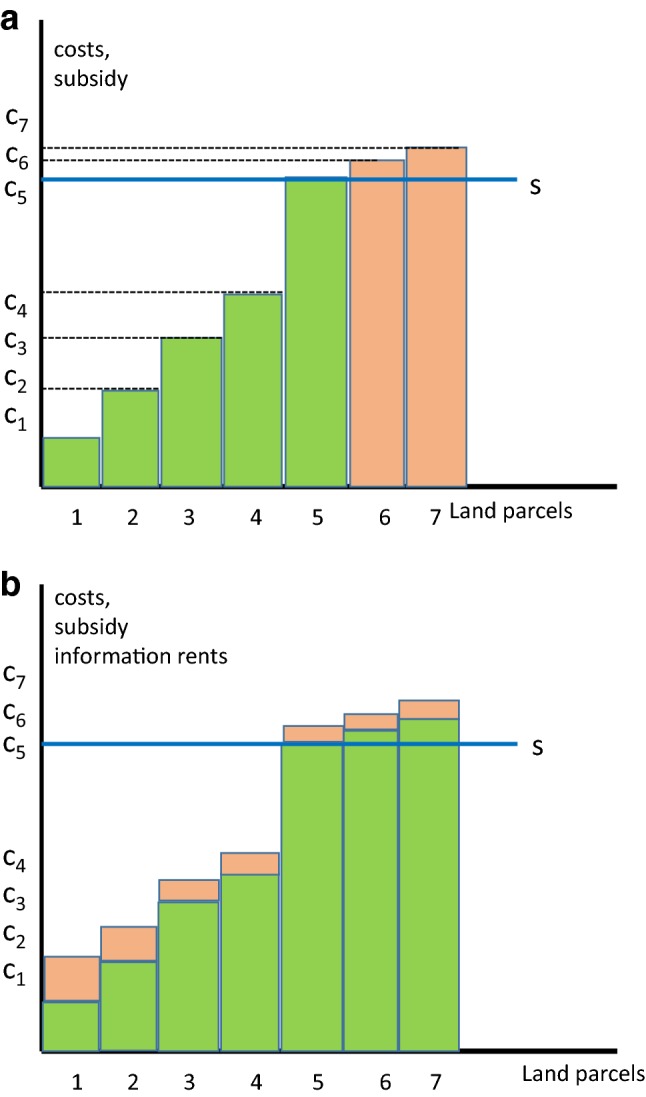
**a** Flat rate payment and participation. **b** Tendering system and participation

Latacz-Lohmann and van der Hamsvoort (1997) demonstrate the usefulness of tendering over flat rate policy. Figure 2a, b uses tendering to establish our argument in favor of performance-based instruments in an intuitive way. Consider an area-based support payment for participation in the national agri-environmental scheme. The payment compensates for the costs of taking measures to reduce runoff (such as buffer strips, gypsum, catch crops, or structural liming). Let the annual government budget be *G* and the area payment be *s* per ha. Suppose for simplicity that each farmer supplies a field parcel of size one hectare (denoted by *i*) to the program and implements some of the listed water quality measures on this parcel. The costs of these measures, *c*_*i*_, differ between farms. Figure 2a orders the submitted parcels from the least expensive to the most expensive. The horizontal axis measures the number of parcels, and the vertical axis measures the costs and the subsidy.

In Fig. 2a, parcel 5 is the last enrolled in the program, and s*5 = *G* (the budget is exhausted). The last parcel (5) receives a compensation that exactly matches its farmer’s opportunity costs but the others receive “overcompensation” (area between the pillars and the horizontal flat rate *s*), because their costs are lower than the payment. This overcompensation is information rent, as it results from the authorities’ incomplete information on farmers’ costs. Because of overcompensation, only 5 parcels are enrolled in this example, and parcels 6 and 7 remain out of the program.

In environmental tendering, the authorities announce the environmental goals (a reduction nutrient runoff) and invite farmers to offer their fields with assigned water protection measures to the program. While under a discriminatory tendering, each enrolled field parcel receives exactly its bid; under a uniform tendering, a uniform compensation is paid for all winning parcels (Romstad et al. 2012). Under tendering, the challenge of asymmetric information regarding the effects of implementing particular measures at different fields remains, but for cases with similar effectiveness of measures, this leads to selecting the lowest cost options.

Figure 2b illustrates an outcome of a discriminatory tendering system under the farmers’ original opportunity costs and the original conservation budget. The sum of the cost pillar and new light red pillar indicates the size of the bid in each parcel. This sum indicates the bid curve associated with supplied parcels. Thanks to the light red pillars, it is located above the true cost curve but starts well below the flat rate *s* and surpasses it at some point. As farmers are paid according to their bids, the tendering system reduces information rents to farmers much below that of the flat rate subsidy (the reduction of information rent is the white area above the bids and below *s*). As the government saves money, more fields can be allocated in the program. In Fig. 2b, additional parcels 6 and 7 are enrolled, indicating that water protection effort increases due to more efficient use of the original government budget money.

Both tendering and flat rate policies can be improved by using a performance-based approach, such as environmental benefit indexes (EBIs), which assess the environmental performance of the chosen measures. An EBI is simply a number, scaled, for instance, between 0 and 1 or 0 and 100. An EBI is a product of chosen features drawing on their modeled impacts on water quality and weighted by their relative contribution to it. For instance, such features include slopes of fields, sizes of the buffer strips, or soil phosphorus reserves. How well the constructed EBI describes factors determining nutrient runoff depends directly on the state of scientific knowledge. EBIs also help to differentiate fixed payment rates and target more efficiently environmental outcomes, as is done in the Conservation Reserve Program (CRP) (Hellerstein 2017).

In the Baltic Sea region, Finland arranged a tendering pilot with an EBI focusing on a reduction of phosphorus loads. The EBI was constructed using three features: soil phosphorus contents, slopes of fields, and distances to water ways. Field parcels were enrolled in the program according to the ratio of an EBI to bid. Information rents turned out to be very low, approximately 5–10% of the payments (see Iho et al. 2014). Despite a good experience, Finland has not adopted such a tendering system. One obstacle for introducing these performance-based incentives is that payments for the amount of reduced loads are not feasible under the present EU regulation, which allows only compensation for the cost. This refusal to accept incentives, which are important for the performance-based instruments, is artificial and mistaken: as Fig. 2a shows, the area payment equals the conservation costs of only the last parcel, and other parcels receive information rent. This rigid and unfounded regulation has prevented the introduction of modern instruments, such as tendering systems, to promote efficiency and the targeting of environmental protection efforts.

Interestingly, there is an ongoing reform to change the CAP payment system to better facilitate country-specific schemes (European Commission 2019) and offer grants as incentives to farmers to adopt environmental and climate friendly practices, going beyond the costs incurred or the income foregone, but still conforming to least-trade-distorting rules (green box) set by the WTO.

We emphasize the need to change the present rigid EU regulation to facilitate modern, incentive-based, and performance-oriented agri-environmental policy instruments in the process toward the post-2020 CAP.

### Livestock production and manure: Policies for semi-nonpoint polluters

Livestock production provides a challenge of its own. The increasing size of animal farms and high regional concentration of farms create pressure on water quality but may also provide possibilities for new innovative solutions (Schnitkey and Miranda 1993; Harrison et al. 1996; Aillery et al. 2005). A livestock farm has barns and manure storages with possible leakages, and they are considered point sources from a policy angle. Cultivation of fodder, crop, and pasture are in turn sources of diffuse loads but manure complicates cultivation and land use in livestock farms.

Manure is kept in storages that may leak, sometimes with detrimental impacts. When manure is used as a fertilizer and spread on the soil surface, nutrients may easily be released to water ways. Water protection can be promoted by renovating all manure storages, facilitating better timing and utilization of the manure applications, and shifting to more efficient spreading technologies. Jansson et al. (in the present volume) find large differences in the load reductions from manure investments between the countries. While the potential is modest in Denmark, which already has mandatory and high requirements regarding utilization of nitrogen in manure, the effects are higher in Sweden, Finland, Poland, and the three Baltic states. Sufficient storage capacity is important for farmers to be able to apply the manure on fields during the period when the crops grow and utilize the nutrients from the manure in spring and early summer (Tybirk et al. 2013). The capacity requirements differ between 5 and 10 months in countries around the Baltic Sea. Data are scarce documenting current capacities, but there are options for improvements to facilitate better utilization. The type of slurry also affects the ability to utilize the nutrients. While the share of slurry is 80% in Denmark, this share is 5–10% in Poland. Overall, nearly 50% of the manure in the Baltic Sea region is solid (Tybirk et al. 2013).

Thus, we conclude that there is potential for increasing investment in manure storage to reduce manure leakage cost-effectively.

#### Structural change in livestock production

The development of livestock units and livestock farms has followed a similar pattern in all Baltic Sea countries. From 2005 to 2013, the number of bovine animals has remained approximately the same in all countries, with a slight decrease in total numbers (from 17 869 million livestock units (LSU) to 17 273 million LSU). The number of pigs has decreased in all countries, with a total decline of 14%. Poultry production has increased in almost all countries, with a total increase of approximately 21%. The strongest trend, however, lies in the number of livestock farms: it has decreased by almost 40%, indicating also an increasing farm size and manure concentration (Eurostat 2018). The same structural development will continue, generating increasing pressure for local manure management.

The structural change in livestock farms has important implications for the availability of land for manure applications. Larger farms sizes entail higher risks that manure will be overapplied on the fields closest to animal facilities. Solutions to tackle the problem vary across countries. In many countries, the Nitrate Directive or phosphorus fertilizer limits command that expanding livestock farms have enough manure spreading area. Clearing peat land forest to create fields in Finland has been very detrimental, because new fields have increased deforestation, GHG emissions, and nutrient runoff. Denmark has promoted biogas production, which provides climate benefits but does not alleviate the transportation cost problem unless nutrient separation techniques are adopted.

Thus, current policies have not adapted to the rapid increase in the size of livestock farms (Kauppila et al. 2017), and only few innovations have taken place to solve the manure problem. One reason for this is that livestock farms in most places have not been subjected to tight regulation on manure issues (Jansson et al. in the present volume). Large livestock farms are, however, industrial plants and should be treated as such. Environmental permits provide a tool to promote progress in solving the manure problem. Experience from the US poultry industry provides a good example for how things may evolve. For broiler operations in the Delmarva Peninsula, the US produces more than 600 million birds in a year (Kleinman et al. 2011). Regulation of poultry litter from large farms became more stringent as the states (Delaware, Maryland, and Virginia) responded to water quality issues of the Chesapeake Bay. Delaware, for instance, implemented fully the Delaware Nutrient Management Act in 2007. The recent emergence of brokers and industrial size poultry litter processors represents an innovative response of the industry to tightened regulation^1^. Referring to this experience, we argue the following:

Tighter regulation of livestock production and the processing of manure are important for obtaining new technological solutions and business opportunities.

#### The special challenge of phosphorus

Manure contains nutrients in an uneven agronomic ratio: too much phosphorus relative to nitrogen. Farmers usually target nitrogen fertilization and ignore the applied excess phosphorus, as it does not reduce yields. This creates spatial and temporal challenges for phosphorus policies. Due to increasing transport costs, farmers spread manure closer to the farm center and use mineral fertilizers in more distant fields (Schnitkey and Mirada 1993). Lötjönen et al. (unpubl., results) demonstrate that the same pattern also occurs in the socially optimal solution, but manure is spread less at each distance and for a longer distance than in the private solution. Therefore, the phosphorus content of soil is higher closer to the farm center. Phosphorus from manure accumulates in soil over time, reflecting the annual phosphorus fertilization by manure, the uptake of phosphorus by crops, and soil chemical processes (Iho 2010). Large phosphorus reserves in the soil increase dissolved reactive phosphorus load, which is directly available for algae growth, creating the need of controlling soil P. Soil phosphorus content decreases with distance, but assuming a constant use of mineral phosphorus, it becomes constant. This implies that nutrient runoff differs between parcels in livestock farms.

Unlike in crop production farms, differentiated P policy is optimal, albeit difficult to establish for livestock farms. Furthermore, reducing phosphorus loads will take time, because runoff of dissolved reactive phosphorus depends on soil P, which changes very slowly over time. A target value could be set on the steady-state soil P to define the upper limit on phosphorus fertilization and thereby on manure spreading per hectare (Iho 2010). As the reduction is possible only in the long run, short-term measures, such as gypsum or structural liming, are needed to reduce phosphorus leakage in the short run (Kosenius and Ollikainen 2019). Finally, a tax on mineral fertilizers has an impact on manure spreading, making it more profitable to use manure on more distant fields. Farmers reduce manure use on all locations to make it last for the new locations (Lötjönen et al. unpubl., results).

Increasing farm sizes and regional concentration may provide a starting point for new solutions to the environmental problems related to manure. With strong spatial concentration, it may become profitable to process the manure in industrial-scale facilities, providing a way out of the problems of large-scale animal production. It would also help prevent spatial accumulation of manure nutrients by processing them into forms less expensive to transport and overapplication of the relatively more abundant manure nutrient by decoupling nitrogen and phosphorus fractions. Moreover, it would offer livestock farmers the possibility to focus on the core of their businesses instead of struggling to meet the manure regulations. After all, regulatory issues of manure management are found to be important factors when animal farms are making their relocation decisions (Stirm and St-Pierre 2003). Essentially, this would be a Turn Key solution for farm manure management under wise regulation.

Our suggestion is that promoting industrial-scale treatment of manure in the food processing sector would provide a solution to the multiple environmental challenges created by current manure management in livestock farms.

## Coherence of water and climate-related policies

There are no effective climate policies toward agriculture at the moment, but by 2020, the land use sector will become a part of EU’s climate policy, and rightly, agriculture must take its share in climate mitigation and adaptation efforts. In the climate context, agriculture presents both a problem and a solution. GHG emissions from cultivation, soil and animals are considerable and boost global warming, while nutrient runoff has regional impacts on water quality. Agriculture is a solution when reducing emissions and especially sequestering carbon in soils. Not all measures, however, promote both water quality and climate targets. It is important to ensure coherence between climate and water policies targeting agriculture.

Crop rotation with legumes is beneficial for both climate and water ecosystems (Lötjönen and Ollikainen 2017). Legumes help to reduce the use of mineral fertilizers by fixing nitrogen from the air and providing the residual fertilization effect for crops to be grown the following year. Legumes, buffer strips, and crop rotation promote simultaneously both climate and water goals. A constraining factor is limited demand for legumes, implying that it is well-suited to livestock production areas only. Here, the increasing ambition of the EU’s legume policies would promote both water and climate targets.

Introducing climate policies to livestock production provides a challenge, as they have only few possibilities to reduce GHG emissions. For instance, both manure management and diet make only minor contributions. The main source of GHG emissions is methane emissions from animals, and currently the only known means to reduce emission from animals is to reduce their number. Water policies in contrast target a larger set of choices and provide livestock farms a leeway to adjust cultivation and manure handling without reducing the number of animals. Thus, climate policy hits more strongly on the profits of livestock farms (Lötjönen et al. unpubl., results). The water quality targets must not be compromised when climate policy is given more attention (Nainggolan et al. 2018). Thus, we emphasize the following:

Introducing the much required climate policies to agriculture must be made with full coherence to water quality targets requiring novel performance-based type instruments for agriculture.

## Incentives for innovation

Agriculture in the Baltic Sea region needs higher productivity, active climate mitigation, and better performance in promoting water quality. Growing population in the catchment implies an increased pressure on surface water quality. Society must promote long-term solutions for all these issues through improved technologies, production systems and social discoveries. The role of environmental policy for innovation and technological development is therefore important. Three questions regarding innovations are of particular interest: (i) Do current policies provide sufficient incentives for innovation? (ii) If not, how can the incentives be improved? (iii) Will the novel technologies be adopted by the intended users?

Markets suffer from under-provisioning of innovations: innovators’ net gains from innovation are small in comparison to the overall gains, because innovations could be copied by other firms (Goulder and Parry 2008). Stringent environmental policies encourage innovation if they imply that polluting becomes more expensive, allow the polluter freely to choose among alternative abatement technologies, and credit the effects of the novel technologies against the firm’s abatement obligations. The choice of policy instrument is crucial for providing incentives for innovation: market-based instruments, such as taxes and tradeable permits, tend to perform better than command-and-control (Requate 2005). If command-and-control is applied, performance-based policies provide stronger incentives for innovation than design standards, i.e., regulation of technology use (Shortle and Horan 2017). Currently, taxes and tradable permits are absent from water quality policies in the Baltic Sea region. Instead, performance standards are widely used for wastewater treatment plants, while design standards and technology-specific subsidies are common in the agricultural sector. Incentives for innovation in abatement technology are weak, especially in the agricultural sector, where the environmental effect of novel technologies that are not subsidized does not increase farm profits.

A comparison of environmental policies toward WWTPs and agriculture provides a good example. Analyzing Swedish environmental policies over 50 years for improved water quality in sewage plants, Häggmark Svensson and Elofsson (2018) show that these policies have increased the number of patents for technologies that reduce nutrient emissions by 40–70% in the years immediately following the introduction of new policy. In a corresponding analysis of agriculture, they find no effect of environmental policy on innovation of nutrient saving technologies, suggesting that policies have been unsuccessful in this regard.

A next challenge is to make farms adopt novel technologies that reduce nutrient emissions to the environment. A study by Konrad et al. (2019), covering Poland, Sweden, Finland, Denmark and Estonia for three nutrient technologies (manure spreading, manure storage, precision fertilizing), confirms the observation from earlier studies that large farms have a higher propensity to adopt new and costly technologies (Lynne 1995; Fuglie and Kascak 2001). This suggests that the ongoing structural development in agriculture may be environmentally beneficial through its effect on technology adoption.

To strengthen innovation as a tool for meeting the Baltic Sea nutrient reduction targets at low cost, an increased use of market- and performance-based policies is needed. A first step could be to apply performance-based policies for larger farms, hence treating them as point sources rather than nonpoint sources, as their having a higher propensity to adopt novel technologies suggests this would enhance both innovation and adoption of novel technologies. The second step would be to develop schemes for nutrient trading, either among point sources (Hautakangas and Ollikainen 2019) or between point and nonpoint sources (Shortle and Horan 2017). The scale of trading would be of central importance for the size of incentives for innovation, as it determines the demand for novel technologies from the users.

Innovation policy must be directly linked to water policies in agriculture in the Baltic Sea region by tighter regulation and use of market-based instruments.

## Voluntary instruments and flexible mechanisms

The analysis has thus far focused on policies or policy instruments that create favorable circumstances for point sources or farmers. The implicit assumption underpinning our discussion has been that once the incentives are set right, the actors will fill their roles for the required effects in the Baltic Sea environment. Voluntary actions by actors may nicely complement the mandatory policies toward point and nonpoint sources.

An interesting form of water policies is to extend ideas of carbon neutrality to water protection issues: companies, cities or private actors could strive for nutrient neutrality by offsetting their loads that remain after abatement. For instance, phosphorus neutrality is a worthwhile goal, as it promotes the quality of coastal waters. A municipal waste water treatment plant (WWTP) and city or an industrial point source could offset their loads by buying reduction from another agent that can reduce loads at lower social costs. Moreover, municipal WWTPs could be willing to promote water protection if an equivalent sum of their investment could play for higher reductions elsewhere in the drainage basin. Therefore, pursuing nutrient neutrality should be promoted by creating transparent and clear systems for nutrient compensations. To provide an example, four Finnish WWTPs located on the coast of the Gulf of Finland compensated for their P loads by investing in abatement in Vitebsk, Belorussia.

More importantly, nutrient compensations may have a much higher status in the future. Water protection within the EU is unified by the Water Framework Directive (WFD). The recent Weser ruling of the European Court of Justice (C-461/13) strengthened the legal status of WFD-specific water quality standards, which will be reflected in environmental permitting processes also around the Baltic Sea. Under a strict interpretation, an environmental permit cannot be given to an economic activity if it increases the pollution of elements critical to water quality standards.

To prevent the emergence of unintended constraints from well-intended regulatory changes, some flexibility should be built into environmental instruments. One option is utilizing nutrient offsets in the permitting process. In our example, the facility would create nutrient credits by decreasing the nutrient loading risk from the livestock facilities it collects the manure from. These credits would be taken into account when determining the net effect of the new facility on nutrient pollution. Similar practices could be used for many economic activities as long as the basic condition is met: the new or expanding economic activity together with the offsetting credit generates a net decrease to total nutrient loading to the respective water body.

We must ensure that the regulation is keeping pace with not only the structural change and the challenges it imposes but also new innovations that help mitigate nutrient loading.

## Recommendations

Our analysis has identified weaknesses and possibilities for improvements in Baltic Sea protection policies. For point sources, the key weaknesses include too lax regulatory policies toward WWTPs and missing incentives for developing new and novel abatement solutions, for instance, promoting a circular economy. For nonpoint sources, current CAP policies prevent using performance-based policies. Inefficient regulation of livestock farming and missing incentives for promoting technological developments belong to other key weaknesses. The invented novel policy principles and instruments facilitate improving the environmental and economic efficiency of Baltic Sea policies. They provide polluting agents with stronger incentives to protect the sea, to promote environmentally friendly innovations, and to engage voluntary citizens in useful work for the Baltic Sea, and they constitute a sound standpoint to meet the challenges that climate change brings to the Baltic Sea region in coherence with water policy requirements.

The research community can now provide key solutions to reduce loading and fit the long-term economic growth to the ecological limits of the sea with all stakeholders engaged to determined actions. A Baltic Sea Socioeconomic Action Plan is called for to systematically update and strengthen nutrient policies in the Baltic Sea region countries. As the first steps toward developing this plan, we suggest that the following features should be the backbone of such a plan.

1. Cost-effective abatement with equalization of marginal abatement cost should be the guiding principle of nutrient policies toward point and nonpoint sources, because this principle reflects best the technological and economic possibilities to reduce loads the most.
2. Both the EU’s Urban Waste Water Directive and HELCOM recommendations are inattentive relative to current abatement possibilities and costs in WWTPs. They should therefore be scaled up accordingly to promote cost-effective abatement.
3. The rigid EU CAP policy toward agriculture should be changed to facilitate modern, incentive-based, and performance-oriented agri-environmental policy instruments instead of the current measure-based approach.
4. Tighter regulation of large livestock farms and promoting industrial-scale treatment of manure in the food processing sector provide possibilities for new technological solutions and business opportunities.
5. Promoting industrial-scale treatment of manure by vertically integrating the food processing sector would provide one possible solution to farm manure management.
6. There are ample possibilities to create coherent nutrient and climate mitigation measures in agriculture that should be utilized.
7. Regulation must keep pace with economic development and tightening environmental standards by utilizing flexible and innovative instruments, such as nutrient offsets.

## Footnotes

1 https://www.perduefarms.com/news/press-releases/perdue-expands-nutrient-recycling/ and https://www.voanews.com/a/mayland-chicken-manure-global-environmental-polution/3050598.html.
